# Assessing Systems of Care for US Children with Epilepsy/Seizure Disorder

**DOI:** 10.1155/2013/825824

**Published:** 2013-10-21

**Authors:** Mary Kay Kenney, Marie Mann

**Affiliations:** Maternal and Child Health Bureau, Health Resources and Services Administration, U.S. Department of Health and Human Services, 5600 Fishers Lane, Rm 18-41, Rockville, MD 20857, USA

## Abstract

*Background*. The proportion of US children with special health care needs (CSHCN) with epilepsy/seizure disorder who receive care in high-quality health service systems was examined. *Methodology*. We analyzed data for 40,242 CSHCN from the 2009-2010 National Survey of CSHCN and compared CSHCN with epilepsy/seizure disorder to CSHCN without epilepsy/seizure disorder. Measures included attainment rates for 6 federal quality indicators with comparisons conducted using chi square and logistic regression methods. In addition, CSHCN with epilepsy/seizure disorder were compared to CSHCN without epilepsy/seizure disorder on the basis of 14 unmet health care needs. *Results*. Lower attainment rates for receiving comprehensive care in a medical home and easily accessible community-based services were found for CSHCN with epilepsy/seizure disorder versus CSHCN without epilepsy/seizure disorder (medical home: 32% versus 43%; accessible community-based services: 50% versus 66%, resp.) in unadjusted analyses. Lower adjusted odds for these indicators as well as greater unmet need for specialists, dentistry, prescriptions, therapies, and mental health care were also found for CSHCN with epilepsy/seizure disorder. *Conclusions*. Further efforts are needed to improve attainment of high-quality health care services for CSHCN with epilepsy/seizure disorders.

## 1. Introduction

Childhood seizures are often accompanied by cognitive and behavioral deficits which may be induced or exacerbated by the seizure disorder and vary based on underlying neuropathology (e.g., perinatal brain injury and congenital central nervous system malformation), seizure type, age of onset, psychosocial problems, and treatment side effects [1]. Recent research has increased our understanding of the range of difficulties accompanying a diagnosis of childhood epilepsy/seizure disorder. Children with currently reported epilepsy/seizure disorder were significantly more likely than those never diagnosed to experience depression, anxiety, attention deficit/hyperactivity disorder, conduct problems, learning disability, developmental delay, and autism/autism spectrum disorder [2]. They had greater risk of experiencing a range of physical health comorbidities including hearing or vision problems, asthma, headaches, allergies, ear infections, and poor oral health. Functionally, children with current epilepsy/seizure disorder were more likely to have limited activity, grade repetition, school problems, and low social competence compared with children never diagnosed. High levels of parent aggravation were also reported. Depending on the drug treatment involved, medication side effects can include double vision, weight gain, hyperactivity, sleep disturbances, irritability, gum dysplasia, and mood changes [3, 4].

 The Institute of Medicine (IOM) has addressed improving access to health care for people with epilepsy, taking note of recent evidence of disparities in access to specialized epilepsy care among low SES, racial/ethnic minority populations, and the uninsured [5]. More frequent emergency room visits, higher hospitalization rates, and lower likelihood of neurologist visits among the uninsured and low SES populations have also been noted [6–8]. Very little is known about the healthcare received by children with seizures; however, increased difficulties in obtaining appointments with a neurologist and prolonged wait times for new publicly insured pediatric epilepsy patients have been documented [9, 10]. The IOM has taken note of the many complexities of epilepsy both in its physical impact and accompanying need for appropriate management services delivered through a team-based approach to care [5]. It has outlined the pathway of care for patients with epilepsy/seizure disorder that included roles for both primary care providers and specialists in varying degrees depending on the level of seizure control. The emphasis of their recommendation was on a patient-centered and collaborative approach with strong connections to community resources.

 The purpose of this study was to determine the extent to which children with special health care needs (CSHCN) in USA who have epilepsy/seizure disorder (E/SD) receive the type of care that is consistent with recent recommendations and define the service areas and aspects of comprehensive care that are most lacking in this population. We use as a framework a model system of care that is integrated, comprehensive, coordinated, family centered, and consistent across the life course [11]. The results of this investigation will provide a baseline, measuring the proportion of US children with E/SD who meet six federal core outcomes or quality indicators for children with special health care needs (CSHCN) [12]. Those quality indicators include the following. (1) Families of CSHCN will be engaged in shared decision-making with their primary care provider. (2) CSHCN will receive coordinated, ongoing comprehensive care within a medical home. (3) Families of CSHCN will have adequate private and/or public insurance to pay for the services that they need. (4) CSHCN will be screened early and continuously for special health care needs. (5) Community-based service systems will be organized so that families can use them easily. (6) Youths will receive the services necessary to make transitions to adult life, including adult health care, work, and independence. A comparison group of CSHCN without E/SD will provide context for interpreting the needs of CSHCN with E/SD.

## 2. Methods

### 2.1. Sample and Data Source

 The medical, emotional, and behavioral needs of US children with disabling or handicapping conditions have been addressed by federal legislation enacted over two decades ago. In the Omnibus Budget Reconciliation Act (OBRA) of 1989 the US federal government directed the states to “provide…and promote family-centered, community-based, coordinated care…for children with special health care needs…and facilitate the development of community-based services for such children and their families” [12]. The Health Resources and Services Administration's Maternal and Child Health Bureau administers federal funds that assist the states in providing direct and indirect services to children with special health care needs (CSHCN) and their families. The Maternal and Child Health Bureau developed the framework of six core outcomes or quality care indicators which were described above to guide the states in providing the type of care consistent with the 1989 legislation and recommended by the IOM. The National Survey of Children with Special Health Care Needs (NS-CSHCN) is the survey instrument used to chart the nation's progress in delivering services targeted by the OBRA legislation. 

The National Survey of Children with Special Health Care Needs is a quadrennial random-digit-dialing (land and cell phone lines) survey that was designed to produce national (US) and state-specific prevalence estimates of CSHCN, describe the types of services they need and use, and provide information regarding the system of services available to them [13, 14]. The survey employs a conceptualization of CSHCN that operationally defines a special health care need as a medical, behavioral, or other condition that has lasted, or would be expected to last, at least one year and resulted in at least one of the following: using or needing more medical care, mental health services, or educational services than are generally required by other children of the same age; using or needing prescription medicines; having limitations in the ability to do things other children of the same age do; using or needing special therapy (i.e., physical, occupational, or speech therapy) or assistive devices; or using or needing emotional, developmental, or behavioral treatment or counseling. 

 In 2009-2010, a total of 372,698 children under 18 years old from 196,159 households were screened to identify those with special health care needs. A total of 40,242 detailed CSHCN interviews were collected; at least 750 interviews were conducted in each state and the District of Columbia. CSHCN who have ever been diagnosed (*N* = 1,870) and who are currently diagnosed (*N* = 1,226) with epilepsy/seizure disorder are the subject of this analysis. Further details of the survey methodology are presented elsewhere [13].

### 2.2. Measures and Data Analysis

Below is a description of the questions composing each indicator as well as its scoring.

*Indicator 1: Shared Decision-Making.* This indicator is based on how often during the past 12 months the child's doctor or other health care providers: (1) discussed with the family a range of options to consider for their child's treatment; (2) encouraged the family to ask questions or raise concerns; (3) made it easy to ask questions or raise concerns; and (4) considered and respected what treatment choices the family thought would work best for their child. To positively meet this indicator, responses of “usually” or “always” to all items must be scored. Any response of “never” or “sometimes” to any item would score the indicator in the negative. As with all the indicators, responses to all subcomponents of an indicator were required. All responses of “do not know” or “refuse to answer” were set to missing.

*Indicator 2: Medical Home.* The overall medical home measure is a composite derived from five different subparts based on 19 different survey items. Indicator 2 is attained when the respondent affirmatively answers that their child: (1) has at least one personal doctor or nurse; (2) received family-centered care in the previous 12 months (i.e., health providers usually or always spend enough time with them, listen well, are sensitive to family values and customs, provide needed information, and make family feel like a partner in care); (3) has had no problems getting referrals, when needed; (4) has usual source or sources of sick and well care; (5) receives effective care coordination (i.e., usually or always gets all needed help coordinating care; if applicable, has been very satisfied with the communication between providers and school/daycare and/or between primary provider and other medical providers). To meet this indicator, responses of “yes” or “usually/always” to all items must be scored. Any response of “no” or “seldom/never” to any item would score the indicator in the negative.

*Indicator 3: Consistent and Adequate Health Insurance*. Indicator 3 is attained when the respondent answers that (1) their child was insured at the time of the survey and has had no gaps in coverage in the previous 12 months; (2) their child's health insurance offers benefits that usually or always meet the child's needs; (3) the noncovered insurance charges are usually or always reasonable; and (4) their child's health insurance usually or always allows him or her to see needed providers. To meet this indicator, responses of “yes” or “usually/always” to all items must be scored. Any response of “no” or “seldom/never” to any item would score the indicator in the negative.

*Indicator 4: Early and Continuous Screening*. For Indicator 4, the survey child must have had preventive medical and dental care in the prior 12 months (visits where screening may have occurred). It is based on the following two questions: (1) (During the past 12 months/Since birth), how many times did (survey child) receive a well-child check-up, that is a general check-up, when (he/she) was not sick or injured? and (2) (during the past 12 months), how many times did (survey child) see a dentist for preventive dental care, such as check-ups and dental cleanings? (age 1–17 years). Both questions were scored positively if parents reported that their child had 1 or more preventive medical visits and 1 or more dental visits.

*Indicator 5: Community-Based Services*. To meet Indicator 5, families must have no difficulties or delays in getting services and be only “sometimes” or “never” frustrated in efforts to get services for CSHCN. The survey questions assess a variety of factors pertaining to difficulties families may experience when attempting to receive services for their child: (1) the child's eligibility for the services; (2) the availability of needed services; (3) the existence of waiting lists, backlogs, or other problems getting appointments; (4) cost issues; (5) difficulty getting needed information; or (6) difficulties or delays for any other reason.

*Indicator 6: Transition to Adulthood*. For CSHCN to attain Indicator 6, the following criteria must be met (CSHCN age 12-17 years only): (1) the youth's doctor has discussed each of the following 3 topics with him/her (or parent indicated that such discussions were not needed): transitioning to doctors who treat adults, changing health needs as youth becomes an adult and how to maintain health insurance as an adult and (2) the doctor usually or always encourages the youth to take age-appropriate responsibility for managing his or her own health needs.

Several demographic characteristics were included in these analyses: the child's race/ethnicity (non-Hispanic White, non-Hispanic Black, Hispanic, and non-Hispanic Other); age (0–5, 6–11, 12–17 years) and gender (male/female); household poverty status (≤100%, 101%–200%, 201%–400%, and >400% Federal Poverty Level); language spoken at home (English/Spanish/any other language); highest level of education in the household (<high school, high school, >high school); and urbanicity of home (large metro, medium/small metro, urban nonmetro, urban small town, or rural).

We conducted the analysis of successful attainment of the quality indicators in several steps. First, descriptive measures (Table 1) were used to characterize the unweighted and weighted (estimated) frequency and prevalence (per 1,000) of CSHCN with current epilepsy/seizure disorder (CE/SD = has a current seizure disorder diagnosis) among US CSHCN and unweighted and weighted (estimated) frequency and prevalence (per 1,000) of CSHCN with lifetime epilepsy/seizure disorder (LE/SD = having ever been diagnosed with epilepsy/seizure disorder diagnosis) among US CSHCN. Logistic regression (Table 1) was used to determine the adjusted odds of having CE/SD and LE/SD (among US CSHCN) as a function of the sociodemographic factors described previously. Descriptive methods and chi square tests were also used to determine the percentage of CSHCN with and without CE/SD achieving attainment on each of the six quality indicators and their subcomponents and to determine differences in attainment based on being a CSHCN with and without CE/SD (Table 2).

 Next, we explored the association between the six quality indicators and having/not having CE/SD with and without adjustment for covariates and possible confounders (i.e., sociodemographic variables and comorbid neurologically based conditions) using logistic regression (Table 3). The neurologically based conditions included attention deficit/hyperactivity disorder, brain injury, autism, Down syndrome, cerebral palsy, developmental delay, depression, anxiety, conduct disorder, mental retardation, and migraine. Logistic regression also yielded adjusted prevalence rates and their corresponding standard errors in addition to the odds ratios. 

Finally, for each of the six quality indicators chi square tests were used to determine rate-based differences for each of 14 different unmet needs (routine preventive and specialist care; preventive and other dental care; prescriptions; physical, occupational, and speech therapy; mental health and substance abuse care; hearing, mobility, and communication aids; home health care; durable medical equipment; and eyeglasses or vision care) as a function of meeting/not meeting indicator criteria (Table 4). To determine the effects of epilepsy severity, the results for unmet needs were tested for differences between mild versus moderate versus severe epilepsy. 

 All estimates were statistically weighted to reflect population totals using sample weights. The statistical analysis was conducted using SUDAAN 11.0 (Research Triangle Institute, Research Triangle Park, NC), which accounts for a complex sample design involving stratification, clustering, and multistage sampling. Bonferroni adjustment was used for multiple testing. Various measures of multicollinearity among the independent variables were within acceptable limits (VIF < 1.5, Tolerance > 0.70, and *r* < 0.45).

## 3. Results

 In this nationally representative sample prevalence of CE/SD among US CSHCN was 3.2% (32/1000). Lifetime E/SD among CSHCN was 4.9% (49/1000). No significant variation was noted in CE/SD among US CSHCN as a function of race/ethnicity, age, gender, urbanicity, or household language. Results for lifetime E/SD were similar with the exception of a significant difference in prevalence as a function of medium/small metro status versus large metro status and a lack of significance based on educational status. The odds of having a diagnosis of CE/SD or LE/SD among CSHCN were significantly decreased in families at upper income levels. When some basic sociodemographic characteristics were controlled for, higher income children (>200% FPL) among the population of US CSHCN were approximately 30%–40% less likely to have CE/SD or LE/SD than those at lower income levels (≤200% FPL). CSHCN in families with high school education had 1.8 times greater odds of having CE/SD than children in families with less than high school education. 

Table 2 lists each of the six indicators as well as their subcomponents (along with associated prevalence rates and significance level of the chi square test) for CSHCN that have CE/SD compared to those that do not have CE/SD.

*Indicator 1: Shared Decision-Making*. The estimates for the subcomponents of shared decision-making ranged from approximately 79% (doctors encourage parents to ask questions/raise concerns) to 84% (doctors discuss a range of care/treatment options with parents) for CSHCN with CE/SD. The findings for CSHCN without CE/SD were similar, resulting in no significant difference in the shared decision-making indicator or any of its subcomponents for these two groups of children.

*Indicator 2: Medical Home*. For CSHCN with and without CE/SD the range of attainment for the various subcomponents was very large. Relatively low levels of achievement were noted for receiving effective care coordination (44% and 56% for CSHCN with and without CE/SD, resp.) compared to relatively high attainment scores for having a personal doctor or nurse (97% and 93% for CSHCN with and without CE/SD, resp.). There was a significant difference between CSHCN with and without CE/SD in the percentage with a personal doctor or nurse, those receiving family-centered care (59% for CSHCN with CE/SD and 65% without CE/SD), and the overall indicator of having a medical home (32% for CSHCN with CE/SD and 43% without CE/SD).

*Indicator 3: Consistent and Adequate Health Insurance*. Lower relative levels of attainment were noted for both CSHCN groups with regard to having adequate insurance coverage for the child's needs while the highest levels were achieved for having public or private insurance coverage at the time of the survey. There was no significant difference between CSHCN with and without CE/SD in the overall level of attainment for this indicator (58% and 61%, resp.); however CSHCN with CE/SD were significantly more likely to have public or private insurance at the time of the survey interview (99% for CSHCN with CE/SD and 96% for those without CE/SD).

*Indicator 4: Early and Continuous Screening*. The rank order for the subcomponents of this indicator was the same for both CSHCN with relatively lower levels of attainment for receiving preventive dental care in the past 12 months compared to receiving preventive medical care. However, no significant differences were found for overall attainment or any of its subcomponents between CSHCN with and without CE/SD.

*Indicator 5: Community-Based Services*. A wide range was again noted in attainment rates for the 2 subcomponents of the fifth indicator with relatively lower rates of attainment for having access to all the services (51% and 67% for CSHCN with and without CE/SD, resp.) compared to “never” or only “sometimes” experiencing frustration getting services (78% versus 91% for CSHCN with and without CE/SD, resp.). There was a significant difference between the 2 groups in the overall indicator (50% and 66% for CSHCN with and without CE/SD, resp.) as well as in several subcomponents of the indicator. CSHCN with CE/SD were more likely than those without CE/SD to experience difficulty/delay in getting services due to availability, difficulty in getting appointments, and getting needed information. Families of CSHCN with CE/SD were more likely to experience frustration in obtaining needed services for their children than the families of CSHCN without CE/SD.

*Indicator 6: Transition to Adulthood*. Attainment rates for this indicator were relatively low compared to the other 5 indicators described above. Rates for receiving anticipatory guidance for the transition to adulthood were particularly low for both CSHCN with and without CE/SD (40% and 37%, resp.). Rates for being encouraged to take responsibility for care as an adult were considerably higher (59% and 79%, resp.), particularly for the CSHCN without CE/SD. There was a significant difference between the overall attainment rates for this indicator for CSHCN with CE/SD compared to CSHCN without CE/SD (32% versus 40%, resp.).

Table 3 shows the unadjusted and adjusted odds ratios (adjusted for age, race/ethnicity, gender, income, urbanicity, household language, highest household educational level, and neurologically based comorbid conditions) and adjusted prevalence for attaining each of the indicators as a function of being CSHCN with or without CE/SD. The unadjusted ratios indicate that CSHCN with CE/SD have approximately 40% lower odds of receiving comprehensive, coordinated services within a medical home, 50% lower odds of having the benefit of community-based services that are easy to use, and 30% lower odds of receiving effective transition services (if needed) compared to CSHCN without CE/SD. Adjusting for all covariates eliminated the significant difference between the two groups in the receipt of youth transition services, but the adjusted odds ratios for having access to community-based services that are organized for ease of use were similar to the unadjusted odds ratios. Adjusted odds ratios for receiving comprehensive, coordinated services in a medical home were slightly improved over the unadjusted rates. Controlling for other neurologically based conditions did not alter the pattern of significance.

Table 4 shows parent-reported unmet needs of CSHCN with CE/SD as a function of having met or not met the criteria for each of the six quality indicators. It is apparent from this table that for CSHCN with CE/SD statistically significant reductions in unmet needs were found for at least half of the needs reported by parents of CSHCN with CE/SD: receiving specialist care; preventive dental care; prescription medicine; physical, occupational, and speech therapy; mental health care; home health care; and communication devices. Parents who reported finding services organized for ease of use (Indicator #5) were found to have reductions in unmet need in all of the care areas just described, while parents who reported receiving care through a medical home were found to have reductions in six of the seven areas (the exception being no reduction in unmet need for communication aids/devices). Overall, the greatest reduction in unmet need was for physical, occupational, and speech therapy followed by reduction in unmet needs for preventive dental care. Tests of differences in unmet needs for CSHCN with mild versus moderate versus severe epilepsy did not yield significant findings after Bonferroni adjustment of the *P* values (*P* < 0.003). 

## 4. Discussion

Based on the analyses presented here using the National Survey of Children with Special Health Care Needs, the prevalence of parent-reported current epilepsy/seizure disorder among US CSHCN was 32/1000 (3.2%). Specific consideration of epilepsy in infants and children is important since the incidence rates of epilepsy in this age group are among the highest, and there are specific types of seizures that occur solely or most often in infants and children [15]. Furthermore, the need for care coordination has been established previously. Specifically, it has been shown that children with seizure disorders have the highest levels of parent-reported need for care coordination [16]. The current analysis presents new findings on the degree to which US children with epilepsy or seizure disorders are receiving care in a system characterized by 6 quality indicators that emphasize comprehensive care coordination and community resource accessibility. We have shown that among the 6 health service quality indicators for CSHCN with CE/SD, the rates for meeting indicator criteria were lowest for receipt of: (1) youth-to-adult health care transition services, (2) coordinated, ongoing, comprehensive care within a medical home, and (3) easily accessible community-based services to address health care needs (Table 2). Furthermore, these 3 quality indicators were the likeliest to show lower attainment rates for CSHCN with CE/SD compared to CSHCN without CE/SD. However, when adjustment for sociodemographic variables was incorporated in logistic regression models (Table 3), only significant differences in the odds of having a medical home and receiving community-based services remained. 

 The survey results reflect a reality encountered by many families attempting to access a health care system that is fragmented, disorganized, and lacking requisite comprehensive services. Limited access to comprehensive, coordinated systems of care is associated with poorer quality of life for children and youth with CE/SD [17]. This effect extends to the families of CSHCN with CE/SD as parents worry not only about their children and changes in family relationships related to the epilepsy, but also about unsatisfactory interactions with their child's health care providers and school personnel and problems accessing community resources for their child's health/academic difficulties [18]. These family stressors can further exacerbate psychosocial and behavioral issues already experienced by the CSHCN with CE/SD [16].

 Additional impressive findings were the significant reductions in unmet needs among CSHCN with CE/SD who met the criteria for receiving coordinated, comprehensive care in a medical home and accessible community services (Table 4). Specifically, CSHCN who met the criteria for Indicators 2 and 5 also showed the greatest number of reductions in unmet needs. Among the CSHCN who met the criteria, the unmet needs for specialist care, preventive dental care, prescriptions, developmental therapies (occupational, physical, and speech), mental health care, and home health care were significantly reduced. Receiving accessible community-based services (Indicator 5) was significantly associated with further reductions in unmet need for communication aids/devices in addition to the above. 

 Thus, it can be seen that for CSHCN with CE/SD these two aspects of care (i.e., comprehensive care and community-based services) may be particularly important for addressing the complex needs that have been repeatedly cited in the literature. For example, the reduction in unmet need for preventive dental care associated with receiving comprehensive care in a medical home and having accessible community services may lower the elevated risk for poor oral health and the increased risk of medication side effects such as gum dysplasia previously cited [2, 3]. Similarly, the considerable reduction in unmet need for occupational, physical, and speech therapy associated with receiving comprehensive care in a medical home and having accessible community services may lower the elevated risk for developmental delay which is also cited [2]. Finally, the cognitive and behavioral deficits to which CSHCN with CE/SD are subject may be more easily addressed in a service model that includes the quality criteria that are the focus of this study [1, 2].

 Comprehensive care with access to mental, oral, and physical health and nonclinical resources for a child with CE/SD requires partnership and cooperation among patients and their families and the multidisciplinary team. It requires more than medical treatment. It requires connection to community resources and coordination across settings, including child care, schools, and community programs. The family-centered medical home is well positioned to provide coordinated, compassionate, family-centered healthcare by forming strong links between the specialist team, primary care provider team, the patient, and his or her family. As complexity increases, there is greater need for a care team to explicitly assume responsibility for coordinating care, which includes determining the coordination needs, creating a plan of care, communicating and exchanging information, facilitating transitions, and connecting and aligning with community resources. The collaboration between primary and subspecialty care providers is an essential element [19]. As defined by the National Quality Forum, care coordination emphasizes shared decision-making and the placement of patient and family at the forefront to ensure that their views, concerns, and needs are taken into consideration [20].

 Future efforts must include supporting the needs of community-based primary care practices to develop the necessary skills and infrastructure to comprehensively coordinate care for children with special health care needs, particularly those children with complex, chronic conditions. Serving as the medical homes for children and youth with E/SD, the practices need to adopt a collaborative team approach and incorporate processes for effective chronic condition management as well as utilize strategies, such as distance learning technology (i.e., telehealth), to connect patients and their families with primary care and subspecialty clinicians, encourage models of comanagement between primary care and subspecialty care providers to improve outcomes, and reduce unnecessary health care use and costs. Work is needed at the systems level to initiate and sustain collaborative relationships. An integrated care delivery model, such as an accountable care organization, can facilitate the communication and coordination across teams that can lead to high-quality and cost-effective care. 

 The current study has several limitations. First, case status for current epilepsy/seizure disorder was based on parent report and may reflect parent fears as much as actual problems or represent a disorder that may actually have been resolved. Secondly, the sample sizes for some subpopulations were small, limiting the types of analyses that could be performed and the stability of some estimates. Finally, the measurement of unmet need and medical home was also based on parent report rather than some objective standard. Despite these limitations, this report is consistent with others in regard to CSHCN having unmet needs possibly due, at least in part, to limitations in comprehensive care coordination and lack of community-based services. 

## 5. Conclusion

 Receiving comprehensive, coordinated care and access to a variety of community-based services provides significant benefit in reducing unmet needs for many different services reported by parents of CSHCN with epilepsy/seizure disorder. Care coordination and community-based services constitute important factors that allow the primary care provider linkages to the important services and specialists that CSHCN with epilepsy require.

## Figures and Tables

**Table 1:** Estimated frequency/prevalence of parent-reported current and lifetime epilepsy/seizure disorder among children with special health care needs; adjusted models of the association between having a current or lifetime parent-reported epilepsy/seizure disorder diagnosis as a function of selected sociodemographic characteristics among CSHCN: National Survey of Children with Special Health Care Needs, USA, 2009-2010.

Sociodemographic characteristic	Current epilepsy/seizure disorder (CE/SD)				Lifetime epilepsy/seizure disorder (LE/SD)			
	Unweighted *N *	Estimated frequency of CE/SD among CSHCN^a^	Prevalence of CE/SD among US CSHCN, per 1,000	Adjusted^†^ model for having CE/SD among US CSHCN AOR (95% CI)	Unweighted *N *	Estimated frequency of LE/SD among CSHCN^a^	Prevalence of LE/SD among US CSHCN, per 1,000	Adjusted^†^ model for having LE/SD among US CSHCN AOR (95% CI)
Total	1,226	240	32		1,870	366	49	
Race								
Non-Hispanic white	827	188	29	reference	1240	287	44	Reference
Non-Hispanic black	147	67	37	1.13 (0.72–1.76)	205	92	51	1.00 (0.74–1.37)
Hispanic	146	59	32	0.96 (0.68–1.36)	246	105	57	1.03 (0.73–1.46)
Non-Hispanic other	107	31	37	1.21 (0.81–1.80)	179	53	63	1.36 (0.99–1.88)
Age (Yrs)								
1–5	240	76	33	reference	348	114	50	Reference
6–11	467	131	31	0.95 (0.70–1.30)	706	203	47	0.99 (0.78–1.28)
12–17	519	138	31	0.99 (0.77–1.27)	816	220	49	1.07 (0.86–1.34)
Gender								
Male	709	202	31	0.97 (0.78–1.22)	1,109	324	49	1.04 (0.87–1.23)
Female	514	144	32	reference	758	214	48	Reference
Income (FPL)^b^								
≤100%	296	101	41	reference	458	162	65	Reference
101–200%	253	88	36	0.86 (0.54–1.36)	402	128	53	0.82 (0.58–1.14)
201–400%	356	82	26	**0.63 (0.44**–**0.90)**	535	139	44	**0.71 (0.53**–**0.94)**
400+%	321	75	24	**0.61 (0.41**–**0.89)**	475	109	36	**0.59 (0.43**–**0.80)**
Education								
Less than high school	71	33	27	reference	128	65	53	Reference
High school	223	96	43	**1.80 (1.09**–**3.00)**	337	142	64	1.45 (0.99–2.12)
More than high school	932	217	28	1.45 (0.91–2.32)	1,406	331	43	1.20 (0.84–1.71)
Urbanicity								
Large metro	714	242	31	reference	1,103	373	48	Reference
Medium/small metro	111	27	26	0.84 (0.60–1.16)	158	36	35	**0.74 (0.56**–**0.98)**
Urban nonmetro	182	39	34	1.03 (0.76–1.39)	282	64	56	1.12 (0.86–1.47)
Urban small town	108	24	36	1.06 (0.74–1.53)	162	38	56	1.12 (0.83–1.50)
Rural	107	13	28	0.83 (0.56–1.22)	158	24	51	1.01 (0.71–1.44)
Household language								
English	1169	316	31	reference	1759	478	46	Reference
Spanish	43	23	37	1.15 (0.63–2.09)	90	49	78	1.53 (0.97–2.42)
Other	14	6	47	1.45 (0.68–3.13)	21	10	85	1.72 (0.78–3.79)

CSHCN: children with special health care needs; CE/SD: current seizure disorder; LE/SD: lifetime seizure disorder; AOR: adjusted odds ratio; CI: confidence interval; FPL: federal poverty level.

^a^Frequencies are expressed in thousands. Population estimates for each category may not equal the total because of rounding error and missing values.

^b^Data are based on the US Department of Health and Human Services poverty guidelines. For households surveyed in 2009 and 2010, 100% of poverty level was defined as $22,050 for a family of 4.

^†^Each model is adjusted for age, race/ethnicity, gender, income, highest level of education in the household, urbanicity, and household language. Significant findings are shown in bold *P* > 0.05. Multiple logistic regression was used to derive adjusted odds ratios (AOR).

**Table 2:** Percentage of children with special health care needs (CSHCN) with and without parent-reported current epilepsy/seizure disorder meeting the criteria for six quality indicators and their subcomponents: National Survey of Children with Special Health Care Needs, USA, 2009-2010.

Quality indicators and their components	Valid *N*	CSHCN with current epilepsy/seizure disorder	CSHCN without current epilepsy/seizure disorder	
		% (SE)	% (SE)	*P *
Indicator 1: shared decision-making^a^				
Child's doctors discussed range of health care/treatment options	40076	84.4 (1.71)	81.6 (0.38)	0.1231
Child's doctors encourage parents to ask questions or raise concerns	40026	78.8 (3.50)	81.5 (0.38)	0.4553
Child's doctors make it easy for parents to ask questions or raise concerns	40070	81.6 (3.58)	86.3 (0.35)	0.2098
Child's doctors respect parent's treatment choices	39992	81.6 (3.56)	84.5 (0.36)	0.4261
Indicator 1 met	39858	68.9 (3.36)	70.3 (0.44)	0.6687

Indicator 2: medical home^b^				
Child has usual source for sick and well care	40082	87.8 (1.50)	89.4 (0.30)	0.1918
Child has usual source for sick care	40146	88.7 (1.46)	90.6 (0.29)	0.7335
Child has usual source for well care	40149	96.4 (0.83)	96.7 (0.17)	0.2849
Child has personal doctor or nurse	40168	**96.6 (0.76)**	**93.0 (0.25)**	**0.0000**
Child has no problems obtaining referrals, when needed	13265	75.5 (2.86)	76.7 (0.74)	0.6871
Child receives family-centered care	39667	**59.2 (3.21)**	**64.8 (0.46)**	**0.0003**
Child's doctors spend enough time with him/her	40014	76.6 (3.51)	77.6 (0.41)	0.0114
Child's doctors listen carefully to his/her parent(s)	40055	84.6 (3.59)	87.8 (0.33)	0.1278
Child's doctors are sensitive to family customs and values	39927	89.2 (1.52)	88.9 (0.33)	0.6456
Child's doctors provided information specific to child's health	40040	73.5 (3.51)	82.7 (0.37)	0.0998
Child's doctors help family feel like partners in care	40063	82.3 (3.62)	87.1 (0.34)	0.7786
Child receives effective care coordination	29831	44.4 (2.95)	56.4 (0.54)	0.3993
Family usually/always receives sufficient help coordinating child's health care	12890	55.9 (4.39)	57.9 (0.83)	0.8207
Family is very satisfied with communication among child's doctors	26401	54.1 (3.25)	63.1 (0.55)	0.0172
Family is very satisfied with doctors' communication to school or programs	11515	48.2 (3.25)	53.3 (0.88)	0.2066
Indicator 2 met	38934	**31.9 (2.52)**	**43.3 (0.46)**	**0.0001**

Indicator 3: consistent and adequate health insurance^c^				
Child had public or private insurance at the time of the interview	40166	**99.1 (0.34)**	**96.4 (0.20)**	**0.0000**
Child had no gaps in coverage during past 12 months	40087	88.6 (3.67)	90.8 (0.29)	0.5552
Child has adequate insurance	38612	61.3 (3.24)	65.8 (0.45)	0.1835
Insurance usually/always meets the child's needs	38865	82.1 (3.59)	87.0 (0.34)	0.1964
Costs not covered by insurance are usually or always reasonable	38734	64.8 (3.29)	71.5 (0.42)	0.0570
Insurance usually or always permits child to see needed providers	38895	86.7 (3.66)	89.6 (0.31)	0.4338
Indicator 3 met	39699	57.9 (3.15)	60.6 (0.46)	0.3939

Indicator 4: early and continuous screening^d^				
Child has received routine preventive medical care in past year	39972	91.5 (1.30)	90.3 (0.27)	0.4018
Child has received routine preventive dental care in past year	39585	82.8 (1.95)	86.0 (0.34)	0.0990
Indicator 4 met	39859	77.4 (2.15)	78.6 (0.39)	0.5709

Indicator 5: community-based services^e^				
Child has no difficulties or delays getting services	39998	**51.0 (3.00)**	**66.6 (0.45)**	**0.0000**
Child has no difficulties or delays due to eligibility	40133	79.7 (3.55)	89.5 (0.32)	0.0119
Child has no difficulties or delays due to availability	40144	**76.5 (3.53)**	**89.2 (0.31)**	**0.0015**
Child has no difficulties or delays due to problems getting appointments	40184	**69.8 (3.39)**	**82.6 (0.37)**	**0.0008**
Child has no difficulties or delays due to cost	40195	75.3 (3.48)	85.4 (0.34)	0.0089
Child has no difficulties or delays due to trouble getting needed information	40191	**84.2 (1.81)**	**91.2 (0.29)**	**0.0001**
Child has no difficulties or delays for any other reason	28170	97.2 (0.78)	97.0 (0.21)	0.7889
Family is never or only sometimes frustrated when trying to get services	40194	**78.2 (3.55)**	**90.6 (0.30)**	**0.0020**
Indicator 5 met	39972	**49.6 (2.97)**	**65.6 (0.45)**	**0.0000**

Indicator 6: transition to adulthood^f^				
Youth receives anticipatory guidance in the transition to adulthood	13663	39.6 (4.16)	36.7 (0.75)	0.4898
Doctors have discussed shift to adult provider, if necessary	4601	57.4 (6.22)	43.3 (1.38)	0.0558
Doctors have discussed future health care needs, if necessary	12354	60.5 (4.30)	59.0 (0.85)	0.7273
Doctors have discussed future insurance needs, if necessary	10409	38.8 (4.33)	35.0 (0.88)	0.3819
Youth has usually or always been encouraged to take responsibility for health care needs	17041	**59.3 (3.69)**	**78.6 (0.60)**	**0.0000**
Indicator 6 met	16214	31.8 (3.79)	40.3 (0.70)	0.0280

Note: for each indicator Bonferroni corrections are applied to determine significance. Findings determined to be significant at the corrected *P* value are bolded; SE: standard error; CSHCN: children with special health care needs.

^a^Families of CSHCN are partners in shared decision-making for the child's optimal health.

^b^CSHCN receive coordinated, ongoing, comprehensive care within a medical home.

^c^CSHCN have consistent and adequate private and/or public insurance to pay for the services they need.

^d^CSHCN are screened early and continuously for special health care needs.

^e^CSHCN can easily access community-based services.

^f^Youth with special health care needs receive the services necessary to make appropriate transitions to adult health care, work, and independence.

**Table 3:** Unadjusted and adjusted odds ratios and adjusted prevalence for meeting criteria for each of the six quality indicators among CSHCN with and without parent-reported epilepsy/seizure disorder: National Survey of Children with Special Health Care Needs, 2009-2010.

Indicator	Unadjusted	Adjusted^a^	Adjusted^a^
	OR (95% CI)	OR (95% CI)	% (SE)
Indicator 1: family shares in decision-making			
Child has epilepsy/seizure disorder	0.93 (0.68–1.27)	1.11 (0.83–1.49)	72.4 (2.85)
Child does not have epilepsy/seizure disorder	Reference	Reference	70.3 (0.44)
Indicator 2: child receives coordinate, ongoing, comprehensive care within a medical home			
Child has epilepsy/seizure disorder	**0.61 (0.49**–**0.77)**	**0.76 (0.61**–**0.96)**	37.7 (2.52)
Child does not have epilepsy/seizure disorder	Reference	Reference	43.2 (0.46)
Indicator 3: family has adequate insurance to pay for the services they need			
Child has epilepsy/seizure disorder	0.89 (0.69–1.15)	0.98 (0.76–1.26)	60.1 (3.02)
Child does not have epilepsy/seizure disorder	Reference	Reference	60.6 (0.46)
Indicator 4: child is screened early and continuously for special health care needs			
Child has epilepsy/seizure disorder	0.93 (0.73–1.19)	0.95 (0.74–1.21)	77.8 (1.99)
Child does not have epilepsy/seizure disorder	Reference	Reference	78.6 (0.39)
Indicator 5: community-based service systems are organized so the family can use them easily			
Child has epilepsy/seizure disorder	**0.52 (0.41**–**0.65)**	**0.65 (0.51**–**0.83)**	55.8 (2.81)
Child does not have epilepsy/seizure disorder	Reference	Reference	65.5 (0.45)
Indicator 6: youth with special health care needs receive services necessary to make the transition to adult life			
Child has epilepsy/seizure disorder	**0.69 (0.49**–**0.98)**	0.87 (0.59–1.28)	36.9 (4.22)
Child does not have epilepsy/seizure disorder	Reference	Reference	40.0 (0.70)

OR: odds ratio; CI: confidence interval; SE: standard error.

Findings significant at *P* < 0.05 are bolded.

^a^Adjusted for age, race/ethnicity, gender, income, urbanicity, household language, household educational level, and other neurologically based comorbid conditions.

**Table 4:** Unadjusted rates of unmet needs for CSHCN with parent-reported diagnosis of current epilepsy/seizure disorder as a function of having met or not met the criteria for each of the six quality indicators: National Survey of Children with Special Health Care Needs, 2009-2010.

Unmet needs	Indicator 1: shared decision-making			Indicator 2: receipt of care through a medical home			Indicator 3: adequate private or public insurance			Indicator 4: early and continuous screening and surveillance			Indicator 5: services organized for ease of use			Indicator 6: effective transition planning for adult health care		
	Indicator 1 Criteria met	Indicator 1 Criteria not met	*P *	Indicator 2 Criteria met	Indicator 2 Criteria not met	*P *	Indicator 3 Criteria met	Indicator 3 Criteria not met	*P *	Indicator 4 Criteria met	Indicator 4 Criteria not met	*P *	Indicator 5 Criteria met	Indicator 5 Criteria not met	*P *	Indicator 6 Criteria met	Indicator 6 Criteria not met	*P *
	% (SE)	% (SE)		% (SE)	% (SE)		% (SE)	% (SE)		% (SE)	% (SE)		% (SE)	% (SE)		% (SE)	% (SE)	
Routine preventive medical care	2.9 (1.03)	3.5 (1.37)	0.71	1.3 (0.61)	3.3 (0.98)	0.08	2.8 (0.96)	3.5 (1.45)	0.71	1.6 (0.79)	8.3 (2.43)	0.01	0.8 (0.43)	5.4 (1.60)	0.005	0.8 (0.77)	5.2 (2.30)	0.08
Specialist care	**1.2** ** (0.40)**	**9.9 ** ** (2.69)**	**<0.001**	**0.6 ** ** (0.47)**	**5.4 ** ** (1.30)**	**<0.001**	2.6 (0.83)	5.6 (1.64)	0.10	3.8 (0.99)	4.1 (1.40)	0.83	**0.6 ** ** (0.40)**	**7.1 ** ** (1.64)**	**<0.001**	8.1 (4.32)	4.5 (1.57)	0.43
Preventive dental care	8.4 (1.37)	16.5 (3.71)	0.03	**5.1 ** ** (1.86)**	**13.6 ** ** (2.00)**	**0.002**	7.4 (1.32)	16.1 (3.03)	0.01	**6.7 ** ** (1.41)**	**26.5 ** ** (3.96)**	**<0.001**	**5.3 ** ** (1.37)**	**17.0 ** ** (2.67)**	**<0.001**	5.4 (2.31)	14.2 (2.98)	0.03
Other dental care	4.2 (1.02)	9.3 (2.98)	0.10	2.3 (1.35)	7.1 (1.56)	0.02	3.6 (1.12)	8.8 (3.20)	0.04	6.0 (1.41)	5.1 (1.61)	0.66	2.6 (1.01)	8.9 (2.07)	0.006	3.2 (2.00)	9.4 (2.53)	0.06
Prescriptions	2.7 (0.92)	7.3 (2.51)	0.08	**0.6 ** ** (0.42)**	**5.0 ** ** (1.30)**	**0.001**	2.6 (0.86)	6.2 (2.04)	0.11	4.4 (1.22)	3.1 (1.34)	0.48	**0.8 ** ** (0.46)**	**7.4 ** ** (1.93)**	**<0.001**	0.6 (0.65)	6.2 (2.04)	0.02
Physical, occupational, speech therapy	**8.4 ** **(1.59)**	**25.5 ** **(4.86)**	**<0.001**	**3.8 ** ** (1.79)**	**17.7 ** ** (2.41)**	**<0.001**	9.6 (1.84)	19.2 (3.41)	0.01	13.2 (2.02)	16.1 (3.41)	0.47	**2.5 ** ** (0.95)**	**24.9 ** ** (3.45)**	**<0.001**	4.9 (2.65)	12.8 (3.01)	0.05
Mental health care	3.7 (1.31)	11.9 (3.15)	0.01	**0.3 ** ** (0.20)**	**8.4 ** ** (1.78)**	**<0.001**	**2.3 ** ** (1.04)**	**11.9 ** ** (2.77)**	**0.001**	7.0 (1.61)	4.4 (1.48)	0.24	**2.3 ** ** (1.16)**	**10.4 ** ** (2.33)**	**0.002**	7.8 (4.14)	9.5 (2.96)	0.73
Substance abuse	0.4 (0.28)	0.4 (0.31)	0.94	0.0 (0.00)	0.6 (0.32)	0.08	0.0 (0.00)	0.8 (0.48)	0.07	0.3 (0.24)	0.7 (0.49)	0.53	0.1 (0.09)	0.7 (0.42)	0.17	0.5 (0.50)	0.0 (0.00)	0.32
Home health care	1.5 (0.50)	6.4 (2.00)	0.01	**0.0 ** ** (0.00)**	**4.5 ** ** (1.03)**	**<0.001**	1.8 (0.60)	4.6 (1.42)	0.06	2.5 (0.76)	4.7 (1.57)	0.21	**0.4 ** ** (0.22)**	**5.6 ** ** (1.38)**	**<0.001**	0.1 (0.10)	6.2 (2.00)	0.00
Eyeglasses/vision care	1.1 (0.57)	1.7 (0.80)	0.52	1.6 (1.10)	0.6 (0.27)	0.37	0.5 (0.22)	1.9 (0.97)	0.15	1.0 (0.50)	2.2 (1.12)	0.31	0.9 (0.69)	1.6 (0.62)	0.46	0.6 (0.45)	2.1 (0.95)	0.14
Hearing aids/hearing care	0.1 (0.10)	2.8 (1.69)	0.12	0.2 (0.22)	1.3 (0.78)	0.19	0.2 (0.12)	2.0 (1.25)	0.15	1.2 (0.69)	0.1 (0.1)	0.10	0.0 (0.00)	1.9 (1.06)	0.08	0.0 (0.00)	1.6 (1.49)	0.28
Mobility aids/devices	4.9 (1.65)	2.5 (0.98)	0.23	6.1 (2.12)	3.3 (1.47)	0.29	4.6 (1.82)	3.7 (1.42)	0.69	2.7 (0.75)	10.8 (5.00)	0.12	5.1 (2.12)	3.5 (1.14)	0.52	4.2 (2.99)	6.8 (3.38)	0.56
Communication aids/devices	3.0 (0.95)	8.0 (3.23)	0.12	2.0 (1.40)	5.4 (1.62)	0.11	2.7 (1.28)	6.7 (1.27)	0.11	4.8 (1.45)	3.4 (1.15)	0.45	**0.47 ** ** (0.19)**	**8.6 ** ** (2.36)**	**<0.001**	0.1 (0.10)	6.4 (2.26)	0.01
Durable medical equipment	2.4 (0.68)	1.3 (0.80)	0.31	0.2 (0.08)	0.6 (0.09)	0.87	1.7 (0.66)	2.3 (0.87)	0.57	1.7 (0.54)	3.2 (1.45)	0.32	0.9 (0.53)	3.1 (0.93)	0.04	1.5 (0.49)	2.1 (0.83)	0.73

SE: standard error.

Bolded items are statistically significant at the Bonferroni-adjusted level of *P* < 0.003.
